# Synthesis and Mechanical Characterisation of an Ultra-Fine Grained Ti-Mg Composite

**DOI:** 10.3390/ma9080688

**Published:** 2016-08-11

**Authors:** Markus Alfreider, Jiwon Jeong, Raphael Esterl, Sang Ho Oh, Daniel Kiener

**Affiliations:** 1 Department of Materials Physics, Montanuniversität Leoben, Jahnstraße 12, Leoben 8700, Austria; markus.alfreider@unileoben.ac.at (M.A.); raphael.esterl@stud.unileoben.ac.at (R.E.); 2 Centre for Integrated Nanostructure Physics, Institute for Basic Science (IBS), Suwon 16419, Korea; jiwonjeong@skku.edu; 3 Department of Energy Science, Sungkyunkwan University (SKKU), Suwon 16419, Korea; sanghooh@skku.edu

**Keywords:** magnesium, titanium, high-pressure torsion (HPT), composite materials, in-situ, microcompression, scanning electron microscopy (SEM)

## Abstract

The importance of lightweight materials such as titanium and magnesium in various technical applications, for example aerospace, medical implants and lightweight construction is well appreciated. The present study is an attempt to combine and improve the mechanical properties of these two materials by forming an ultra-fine grained composite. The material, with a composition of 75 vol% (88.4 wt%) Ti and 25 vol% (11.4 wt%) Mg , was synthesized by powder compression and subsequently deformed by high-pressure torsion. Using focused ion beam machining, miniaturised compression samples were prepared and tested in-situ in a scanning electron microscope to gain insights into local deformation behaviour and mechanical properties of the nanocomposite. Results show outstanding yield strength of around 1250 MPa, which is roughly 200 to 500 MPa higher than literature reports of similar materials. The failure mode of the samples is accounted for by cracking along the phase boundaries.

## 1. Introduction

Titanium (Ti) and magnesium (Mg) are two of the most widely used materials for applications where weight matters, such as in aerospace components [1,2,3] or in medical implants [4,5]. The low strength of Mg is most often the limiting factor for these applications, whereas the higher strength of Ti comes at the cost of higher density compared to the former material. Therefore, combining the advantages of each material seems desirable. However, no binary phases are formed in the Mg-Ti equilibrium phase diagram [6], which makes metallurgical alloying thermodynamically impossible. Earlier attempts focused mainly on enhancing the solubility of Mg in Ti. Zhou et al. [7] achieved an increase of Mg solubility from <0.1 wt% to 1.9 wt% via mechanical alloying, whereas Ward-Close and Partridge [8] used a vapour quenching technique and found an increase of up to 19.5 wt%. However, no mechanical data was reported for this material. Another approach for the combination of both materials is not to form a solid solution, but a microstructural composite. Hassan and Gupta [9] developed a Mg-matrix composite (Mg-5.6 wt% Ti) containing approximately 19 µm large Ti particles and showed an increase in stress measured at the onset of plasticity at 0.2% total strain ($\sigma_{0.2\%}$), from 100 ± 4 MPa to 163 ± 16 MPa as well as ductility (i.e., fracture strain) from 7.7% ± 1.2% to 11.6% ± 1.4%. Lu et al. [10] reported a large increase in the yield strength ($\sigma_{y}$) of Mg-Ti multilayers, which varied inversely with the layer thickness. Another approach is the high-pressure torsion (HPT) technique [11,12] producing a Ti-Mg composite with sub-micron grain size, thereby utilising the Hall-Petch strengthening [13,14] for increased material strength. Edalati et al. [15] applied this technique on a Mg- 33 mol% Ti composite to full saturation and found four different metastable phases within nanograins of 5–10 nm size. In this work, we investigated the mechanical behaviour of similarly produced material. For the evaluation of mechanical properties in combination with the local deformation processes of the composite, microcompression tests [16] were performed on rectangular-shaped samples (pillars), in-situ in a scanning electron microscope (SEM) [17]. This method allows the investigation of microstructural development under load using SEM imaging and a full quantification by simultaneous measurement of force-displacement data, using a modified micro-indentation system.

## 2. Materials and Methods

**Figure 1:**
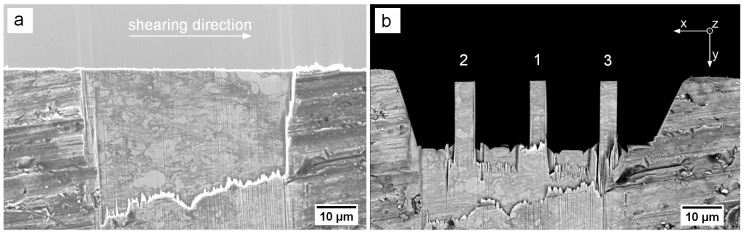
Backscatter electron (BSE) image of a focused ion-beam (FIB) prepared lamella of MgTi depicting (**a**) the microstructure, main shearing direction (**b**) and final micro pillars 1, 2 and 3 before compression testing .

Ti and Mg powders were obtained from Alfa Aesar (Ward Hill, MA, USA) with a purity of 99.5 at% and 99.8 at%, respectively. These powders were sieved using a −500 mesh and mixed in a 75 vol% (88.4 wt%) Ti to 25 vol% (11.4 wt%) Mg proportion, which corresponds to a weight reduction of roughly 15 wt% compared to pure Ti. This composition was chosen for good mechanical comparability with bone considering a loss of Mg by in-vivo dissolution [18,19]. Afterwards, samples were prepared using the two-step HPT technique for compacted powders as described by Bachmaier et al. [20] with a first HPT step of 10 turns and a second HPT step of 100 turns. The two steps are applied perpendicular to each other, for better compaction of the powders, more rapid refinement and more homogeneous deformation of the material in general. From the final disk shaped sample with a diameter of 8 mm and a thickness of 0.8 mm, a 3 × 3 mm^2^ plate was prepared from the outermost radius using diamond wire cutting. This allows an investigation of the volume with the highest applied strain, hence the smallest grain size. One edge of the plate was polished mechanically to a wedge shape of approximately 10° opening angle. No electrolytic methods or water based solutions could be used due to the different solubilities of Mg and Ti and their tendencies to form hydroxides [21,22]. Thereafter, an approximately 10 um thick and 50 um wide section was machined using a focused ion-beam microscope (FIB, LEO 1540 XB, Carl Zeiss, Oberkochen, Germany) as shown in Figure 1a to qualitatively investigate the microstructure. Subsequently, rectangular pillars with a top area of 4 × 4 µm^2^ and an aspect ratio of ∼1:3 were produced using the same FIB station with ion beam currents ranging from 10 nA for coarse cuts to 10 pA for final polishing (Figure 1b). For the sake of discussing deformation behaviour, the samples were numbered in the order shown in Figure 1a. Backscatter electron (BSE) images were recorded before and after testing to examine the influence of the local microstructure on the deformation behaviour. Utilizing the atomic number dependent contrast of Mg and Ti in the BSE images as shown in Figure 1a, an estimation of the average grain size was made using a line intercept method.

Mechanical compression experiments were performed in-situ in an SEM (LEO 982, Carl Zeiss AG, Oberkochen, Germany) using a microindenter system (UNAT-SEM 1, Zwick GmbH & Co. KG, Ulm, Germany) [17] with a maximum applicable force of 300 mN and a conical conductive diamond tip with a flat end diameter of 16 um (Synton-MDP AG, Nidau, Switzerland). Prior to the actual microcompression measurements, a set of 10 elastic loading tests were performed on the lamella to the left of pillar 2 and the right of pillar 3 (Figure 1b) to gauge the system compliance. Stress-strain data was calculated from the measured force-displacement values taking into account the pillar geometry as well as corrections regarding the system compliance and pillar sink-in calculated using Sneddon’s equation [23]. To correlate SEM observations with measured mechanical data, a frame grabbing system was used to sequentially capture SEM images at 1 fps. All tests were operated in displacement-controlled open loop mode with a nominal strain rate of $10^{-3}$ $s^{-1}$.

## 3. Results

**Figure 2:**
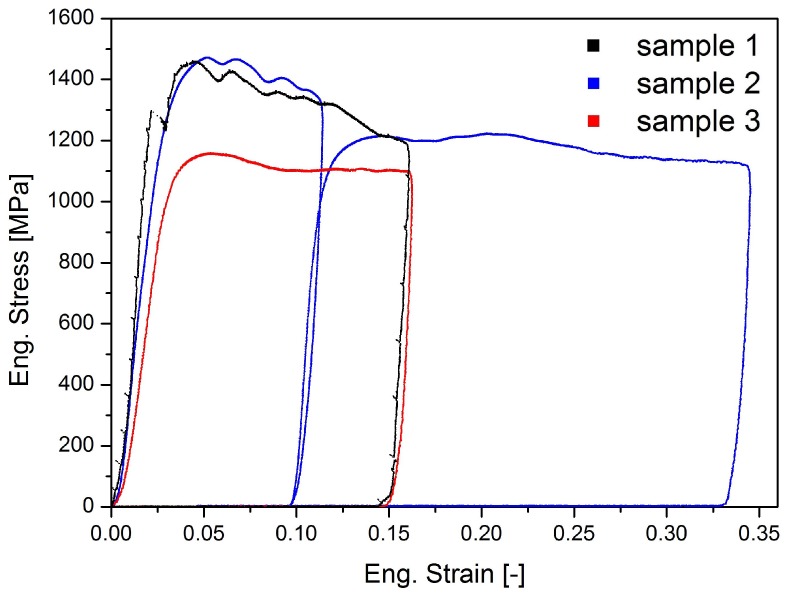
Stress-strain data of all three samples. Sample 2 was compressed twice in order to study larger amounts of deformation.

First, the microstructure was investigated as it plays a crucial role in plastic deformation. The grain sizes measured along the x, y and z directions of square pillars are 749 ± 503 nm, 650 ± 360 nm and 233 ± 105 nm, respectively. The rather large scatter arises due to the limited contrast and resolvability of BSE images for such small grains and potential mechanical mixing. Therefore, the average grain sizes are presumably overestimated. Furthermore, the dimension of the grains perpendicular to the visible plane (z-direction) is smaller, as they are typically elongated in the shearing direction. Generally speaking, the grain sizes of these samples are significantly smaller than 1 um, placing this composite well in the ultra-fine grained (ufg) regime. Although the grain size of our material lies between the grain sizes of identically produced pure Ti (120–150 nm [24,25]) and pure Mg (∼1000 nm [26,27]) samples, it is still considerably higher than that of similar structured composite materials (∼10 nm [15,28]). Therefore, it can be stated that the material was not deformed to fully saturated grain refinement. Figure 2 shows the stress-strain data of all three tested samples. While samples 1 and 2 are similar, sample 3 shows remarkably different behaviour. Nevertheless, some common features are observed, such as the short, strong strain hardening up to an ultimate compressive stress ($R_{m}$) at a total strain of 3%–5% and a subsequent softening leading into a flow state. Details of the testing will be discussed individually.

**Figure 3:**
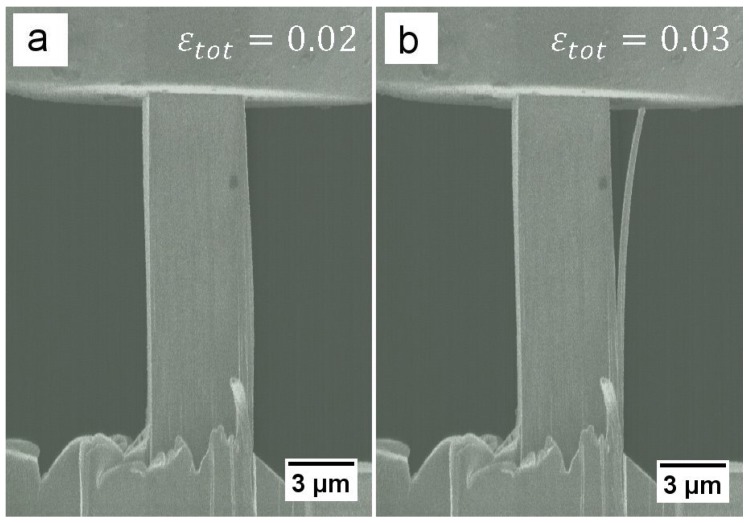
In-situ SEM images of sample 1 during elastic loading (**a**) before and (**b**) after detachment of an FIB induced re-deposition layer.

The stress-strain data of sample 1 (Figure 2, black curve) shows a distinct yielding point at around 1300 MPa followed by a measurable strain burst, which can be explained by the detachment of a re-deposition layer [29,30] that formed during FIB preparation, as shown in Figure 3. This layer is a consequence of the chosen positioning on the lamella. FIB milled materials redeposit on a previously polished surface due to the limited spacing between samples. After yielding, pronounced hardening is evident up to 1460 MPa, followed by a continuous undulated softening, which will be discussed in the following section.

**Figure 4:**
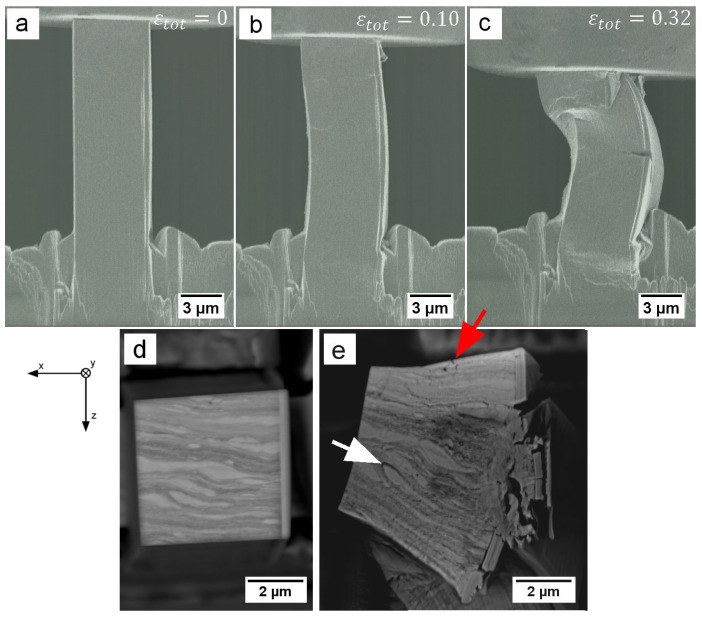
Compression steps of sample 2 (**a**) at contact, (**b**) at a strain of 0.10 during first loading and (**c**) at a strain of 0.32 during second loading. Top view of sample 2 (**d**) before and (**e**) after compression. The white arrow indicates detachment of different phases. The red arrow indicates trans-granular cracking.

Sample 2 shows no detachment of the re-deposition layer and therefore no distinct yield point, but $\sigma_{0.2\%}$ is around 1250 MPa, in good agreement with the previously discussed sample 1. The strong hardening, up to an $R_{m}$ of 1473 MPa and subsequent undulated softening also accord with data from sample 1. Sample 2 was compressed twice in order to study effects at larger strains and subsequent loading. At the second loading (Figure 2, blue curve) the pillar yielded at a lower stress. This is not too surprising, as full unloading took place, which resulted in slight differences in alignment for both cycles. However, the re-loading seems to be in good agreement with the softening trends of pillars 1 and 2. Figure 4 shows in-situ SEM images of the geometrical development of pillar 2 from initial contact to the maximum applied strain in each cycle as well as the top view before and after deformation. A vertical bright line from the re-deposition layer can be observed on the right side of the pillar in all three stages. As show in Figure 4b, deformation leads to slight steps on the surface. According to their shape, these features appear to be boundaries between Mg and Ti grains. Figure 4c depicts the fully compressed state of this pillar with a crack formation in the middle, originated from the high tensile stresses that occur locally in this bent form. The overlaying bending and compression loads on this sample are a result of changing stress states due to non-homogeneous deformation, combined with non-negligible friction forces between pillar and indenter [31], which finally results in crack formation near the top interface in Figure 4c. Notably, this bending does not arise from misalignment, as the determined moduli are around 120 GPa (Table 1), compared to a theoretical value of 96 GPa, assuming a simple rule of mixture. This overestimation of the Young’s moduli is a result of simplifications made in the correction of machine compliance and sink-in calculation. Figure 4d,e show the top view of the previously discussed sample 2 before and after testing, respectively. Some brighter Ti grains are seen to deform slightly, but can still be recognized in comparison with their undeformed shape. Noticeably, part of the deformation results also in detachment of grains and cracking along interphase boundaries (intergranular cracking, white arrow). Crack propagation through grains (intragranular cracking, red arrow) can also be observed to a minor extent in Figure 4.

**Figure 5:**
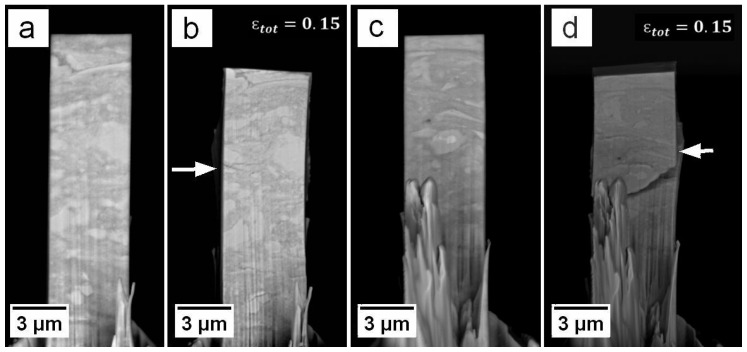
BSE images of sample 3 from front and backside (**a**,**c**) before and (**b**,**d**) after compression to a total strain of 15%. The latter show the formation of a crack (white arrows) propagating through the whole pillar, which seems to be the reason for the different flow behaviour.

In contrast, sample 3 showed a quite different performance (Figure 2, red curve), with a lower $\sigma_{0.2\%}$ of around 1000 MPa, a hardening up to an ultimate compressive stress of 1160 MPa and a nearly constant flow stress level thereafter. The maximum stress for sample 3 is even lower than the yield points of previously discussed samples 1 and 2. Also the prior shown undulated softening behaviour is not visual in this stress-strain data. This flow behaviour might be a result of internal defects, such as voids within the tested volume, presumably due to incomplete adhesion between original powder particles given their strong tendency to form native oxides [21,22]. This is confirmed by in-situ observations of sample 3, shown in Figure 5, where a major crack at mid sample height changes the mechanical behaviour completely.

Figure 5 shows BSE images of sample 3 before and after compression from the front and backside, respectively. In Figure 5b,d, a crack is visible that appeared during uniaxial loading in the absence of pronounced bending and propagated through the whole sample under an angle of ∼45° (white arrows). A major part of the deformation of this pillar was accomplished within the upper sample part, which slipped downwards along the crack plane. Therefore, the pillar also exhibits only negligible lateral thickening during compression from Figure 5a to Figure 5b.

## 4. Discussion

In this part we will firstly discuss the mechanical evolution of the individual pillars as evidenced from our in-situ observations. Moreover, we will give a comparison with similar structured materials documented in literature to point out the outstanding performance of our novel material.

### 4.1. Microcompression

Based on the similar $R_{m}$ and subsequent softening of samples 1 and 2 (Figure 2) in combination with in-situ images, it can be stated that these two samples deform by the same deformation mechanism. Although the exact reason for the undulated softening behaviour is not clear, it is reasonable to assume it being a material response to deformation, as the amount of stress fluctuation (i.e., 60 MPa) and the timely distance (i.e., 20 s) of these oscillations are too large to be related to external influences, such as the electricity network or building vibrations. A possible explanation for this effect is a sequential plastification of softer Mg-rich grains before being blocked by Ti-rich grains that have a higher local yield strength. As this behaviour is depicted in samples 1 and 2, it can also be suspected that the re-deposition layer, which is not present in sample 3, influences the stress-strain data, due to sequential FIB milling. However, no correlation between detachments and changes in yield stress or flow behaviour were observed in-situ. Moreover, in sample 1 detachment occurred, whereas in sample 2 it did not, but the same undulations in the stress-strain data were observed, indicating this being a material behaviour. For comparison, the small strain bursts seen periodically in sample 1 (Figure 2, black curve) are measurement artefacts of electrical nature and therefore do not represent real physical behaviour in the elastic part of the material. Another argument supporting a sequential plastification is shown in Figure 4d,e, where some brighter Ti grains seem to have roughly the same shape before and after compression, implying that they took part in plastic deformation only to a minor extent. Sample 3 depicts a completely different stress-strain behaviour with a 250–300 MPa lower $R_{m}$ and $\sigma_{0.2\%}$ and no undulated softening, but a nearly constant flow level. A very likely explanation for that difference is to assume that a defect within the pillar volume initiated the crack shown in Figure 5b,d, which was the carrier of a major part of the plastic deformation.

### 4.2. Comparison with Literature Data

**Figure 6:**
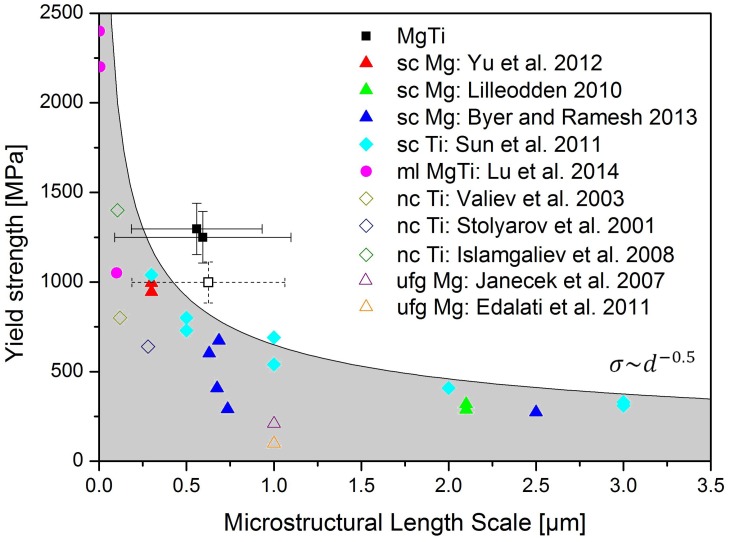
Yield strength comparison with literature data [10,26,34,35,36,37,38,39,40,41]. The microstructural length scale is either the pillar diameter (single-crystalline samples), the grain size (poly-crystalline samples) or the layer thickness (multilayer sample). The open black data point describes the sample containing internal defects. The grey region is bordered by a classical Hall-Petch equation [13,14] with a constant of −0.5.

The yield strengths of samples with dimensions in the sub-micron regime differ from macroscopic investigations [16]. In the case of single-crystalline (sc) specimens with micron-sized geometry, the higher surface to volume ratio leads to dislocation truncation [32] and an associated increase in yield strength, whereas poly-crystalline (pc) samples with grain sizes in the same range increase their strength through dislocation pile-up on grain boundaries [33]. This gives reason to compare the present samples with both, pc samples, as well as sc specimens with miniaturised geometric dimensions. Therefore, it is necessary to take their respective relevant microstructural length scales into account, which is the pillar diameter for sc samples, the grain size for nanocrystalline (nc) and ufg samples, or the layer spacing for multilayer (mL) samples. Figure 6 shows a comparison with literature data, where the filled datapoints describe microcompression experiments on either sc specimens [34,35,36,37] or Mg-Ti mL specimens [10], and the open datapoints describe macroscopic nc [38,39,40] or ufg [26,41] specimens. The sc samples for pure Mg [34,35,36] were all compressed in [0001] direction, whereas the sc Ti samples [37] were compressed along the [11−20] direction. In comparison with these literature values, it is evident that the present samples (Figure 6, filled black datapoints) reveal yield stresses ∼250–500 MPa higher than previously reported materials with comparable dimensions or microstructural length scales.

The only pillar with a lower yielding point is the one previously discussed to contain internal defects (sample 3, Figure 6 open black datapoint). It is evident from the literature, that the sc data generally lies above that of pc materials for the same microstructural length scale. Considering a classical Hall-Petch behaviour ($\sigma ∼d^{-0.5}$), it can be shown that our material exceeds this estimation. The major part of the observed difference between measured data and literature data is presumably a result of the increase in dislocation density, introduced through the HPT process [11]. Additional factors, to be addressed in further investigations, are the currently unknown properties of the grain boundaries and possible contributions from solution strengthening. As both Mg and Ti are highly reactive with oxygen [21,22], it is safe to assume that some of the interphase boundaries also contain fragments of deformed passivation layers from the starting powders. This argument can be supported by the fact that cracking of the specimen seems to occur mostly along these boundaries (Figure 4), which could be a result of poor adhesion between compressed oxide layers. Furthermore, strengthening can arise from the forced mechanical alloying during HPT, causing supersaturated solid-solutions between Ti and Mg.

## 5. Conclusions

We were successful in producing a composite sample of Ti-25 vol% Mg using a two-step HPT process starting from powders, which was examined afterwards though applying in-situ SEM microcompression testing. The yield strengths were found to be significantly higher than for comparable single-crystalline, nano-crystalline or multilayered structures. A constraint for practical applications could be crack formation during HPT processing. However, deformation induced cracking occurred mostly along oxidized interphase boundaries, which can be avoided in future by an annealing step in a reducing atmosphere before powder compaction. 

## Figures and Tables

**Table 1:** Summary of structural and mechanical data: grain size *d*, yield strength $\sigma_{y}$/ $\sigma_{0.2\%}$, ultimate compressive strength $R_{m}$, and Young’s modulus *E*. For sample 1 the strain burst event evident in Figure 2 is used as yield point, for the other samples the stress at 0.2% plastic strain was used. Young’s moduli (*E*) derived from the unloading slope depict a small standard deviation individually but noticeable disparity amongst themselves. Deviations were calculated using error propagation formalisms, which take into account measured data noise and image noise.

#	Yield Strength $\sigma_{y}$/ $\sigma_{0.2\%}$ (MPa)	Ultimate Compressive Strength $R_{m}$ (MPa)	Young’s Modulus *E* (GPa)
sample 1	1250 ± 144	1473 ± 214	124 ± 0.66
sample 2	1297 ± 143	1460 ± 161	109 ± 0.61
sample 3	998 ± 114	1160 ± 134	139 ± 0.63
